# Supplementary material for: A modular approach to integrating multiple data sources into real-time clinical prediction for pediatric diarrhea
Source: eLife. 2021 Feb 2;10:e63009. doi: 10.7554/eLife.63009 (PMC7853717; doi:10.7554/eLife.63009)
Supplement: Table 3—source data 1. [file elife-63009-table3-data1.docx]

|  |  | | Model | |  | |
| --- | --- | --- | --- | --- | --- | --- |
|  | Pre-test * PresPtnt | | PresPtnt * Seasonal | | PresPtnt | |
| Pathogen(s) | Sp.=0.90 | Sp.=0.95 | Sp.=0.90 | Sp.=0.95 | Sp.=0.90 | Sp.=0.95 |
| ST-ETEC | 5 | 3 | 4 | 2 | 5 | 2 |
| Shigella/EIEC | 4 | 1 | 5 | 0 | 4 | 1 |
| Cryptosporidium | 4 | 2 | 3 | 2 | 9 | 5 |
| Cryptosporidium+Rotavirus | 3 | 2 | 2 | 1 | 3 | 3 |
| H. pylori+Rotavirus | 3 | 2 | 3 | 2 | 1 | 0 |
| Rotavirus+TEPEC | 1 | 1 | 1 | 1 | 1 | 0 |
| C. jejuni/C. coli+Rotavirus | 1 | 1 | 1 | 1 | 1 | 1 |
| TEPEC | 1 | 1 | 1 | 1 | 1 | 1 |
| Adenovirus 40/41+Shigella/EIEC | 1 | 0 | 0 | 0 | 1 | 0 |
| Rotavirus+ST-ETEC | 1 | 1 | 2 | 0 | 0 | 0 |
| Rotavirus+Shigella/EIEC | 1 | 1 | 1 | 0 | 0 | 0 |
| salmonella | 1 | 0 | 0 | 0 | 0 | 0 |
| Astrovirus+TEPEC | 1 | 0 | 0 | 0 | 0 | 0 |
| Norvirus GII+Shigella/EIEC | 1 | 1 | 1 | 1 | 0 | 0 |
| Astrovirus+Shigella/EIEC | 0 | 0 | 1 | 0 | 0 | 0 |
| C. jejuni/C. coli+Crypto. | 0 | 0 | 0 | 0 | 1 | 0 |
| Cryptosporidium+ST-ETEC | 0 | 0 | 0 | 0 | 1 | 0 |
| Adenovirus 40/41+ST-ETEC | 0 | 0 | 0 | 0 | 1 | 1 |
| Adenovirus 40/41+Crypto. | 1 | 0 | 0 | 0 | 1 | 1 |
| H. pylori+Shigella+V. cholerae | 0 | 0 | 0 | 0 | 1 | 0 |
|  | 29 | 16 | 25 | 11 | 31 | 15 |

**Table 3-source data 1.** Frequency table of pathogens in which the post-test odds formulation with varying specifity (Sp.) chosen have false positives.
